# Supplementary material for: Structure and proposed DNA delivery mechanism of a marine roseophage
Source: Nat Commun. 2023 Jun 17;14:3609. doi: 10.1038/s41467-023-39220-y (PMC10276861; doi:10.1038/s41467-023-39220-y)
Supplement: Supplementary file 1 — Supplementary Information [file 41467_2023_39220_MOESM1_ESM.pdf]

Supplementary Information for

Structure and proposed DNA delivery mechanism of a marine roseophage

Yang Huang [1,2,#], Hui Sun [1,2,#], Shuzhen Wei [3,#], Lanlan Cai [4], Liqin Liu [1,2], Yanan Jiang [1,2], Jiabao Xin [1,2], Zhenqin Chen [1,2], Yuqiong Que [1,2], Zhibo Kong [1,2], Tingting Li [1,2], Hai Yu [1,2], Jun Zhang [1,2], Ying Gu [1,2], Qingbing Zheng [1,2,*], Shaowei Li [1,2,*], Rui Zhang [3,5,*], Ningshao Xia [1,2,6,*]

[1] State Key Laboratory of Molecular Vaccinology and Molecular Diagnostics, School of Public Health, School of Life Sciences, Xiamen University, Xiamen 361102, China

[2] National Institute of Diagnostics and Vaccine Development in Infectious Diseases, Xiamen University, Xiamen 361102, China

[3] State Key Laboratory of Marine Environmental Science, Fujian Key Laboratory of Marine Carbon Sequestration, College of Ocean and Earth Sciences, Xiamen University, Xiamen 361102, China

[4] Department of Ocean Science, The Hong Kong University of Science and Technology, Hong Kong, China

[5] Institute for Advanced Study, Shenzhen University, Shenzhen 518060, China

[6] Research Unit of Frontier Technology of Structural Vaccinology, Chinese Academy of Medical Sciences, Xiamen 361102, China

- Corresponding authors: abing0811@xmu.edu.cn (Q.Z.), shaowei@xmu.edu.cn (S.L.), ruizhang@xmu.edu.cn (R.Z.) and nsxia@xmu.edu.cn (N.X.).

# These authors contributed equally.

This PDF file includes:

Supplementary Figures 1-16

Supplementary Tables 1-2

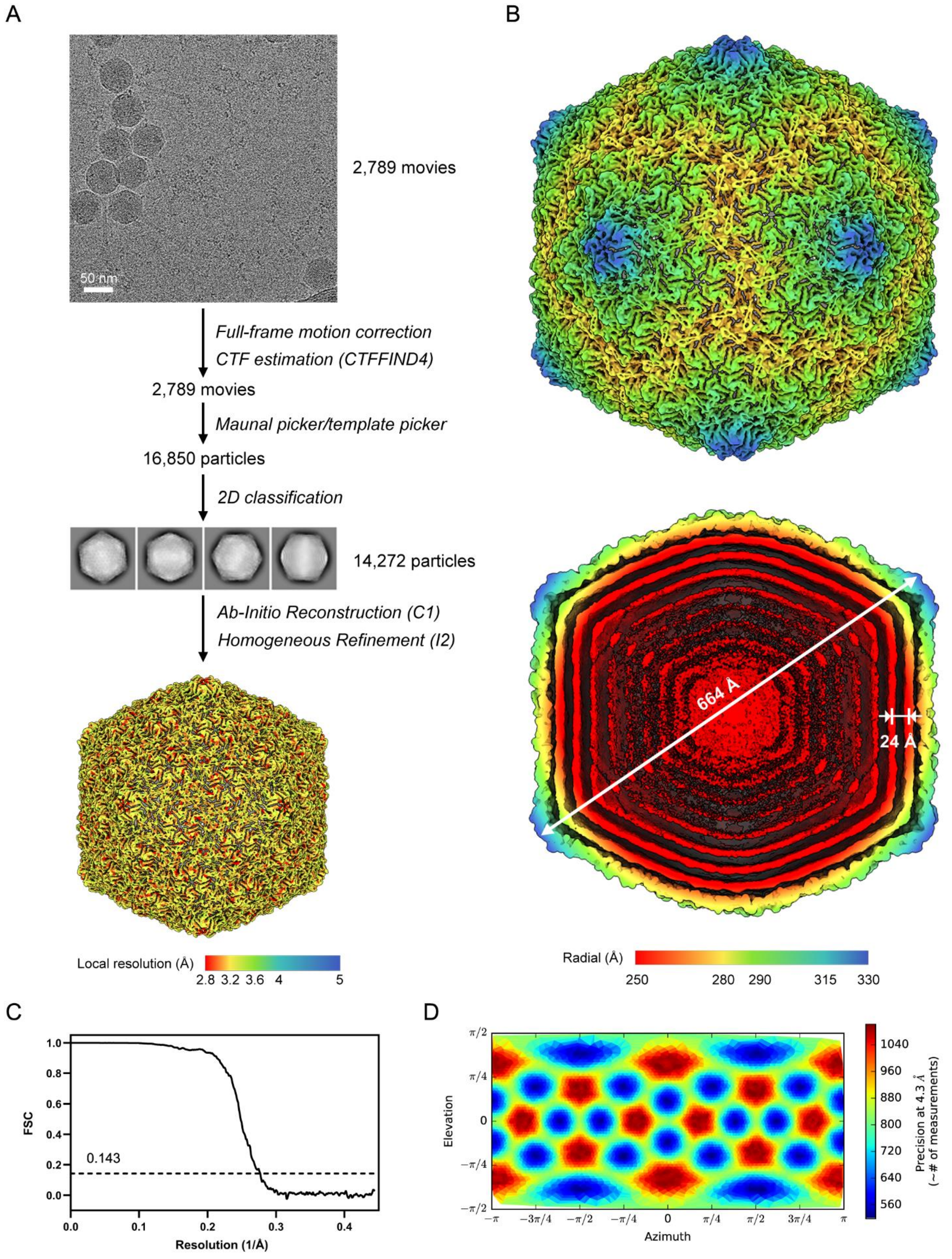

Supplementary Fig. 1. Workflow for the icosahedral reconstruction of the R4C capsid. A. Flowchart for image processing and refinement of the R4C capsid. **B.** Radially coloured density map of the R4C capsid (upper panel) and its central section (lower panel) showing the internal genome densities. The largest capsid diameter (664 Å) and the distance between the recognized genome layers (24 Å) are labelled. **C.** Fourier shell correlation (FSC) curves of 3D reconstructions of the R4C capsid. **D.** The angular distribution of particles used in the final reconstruction.

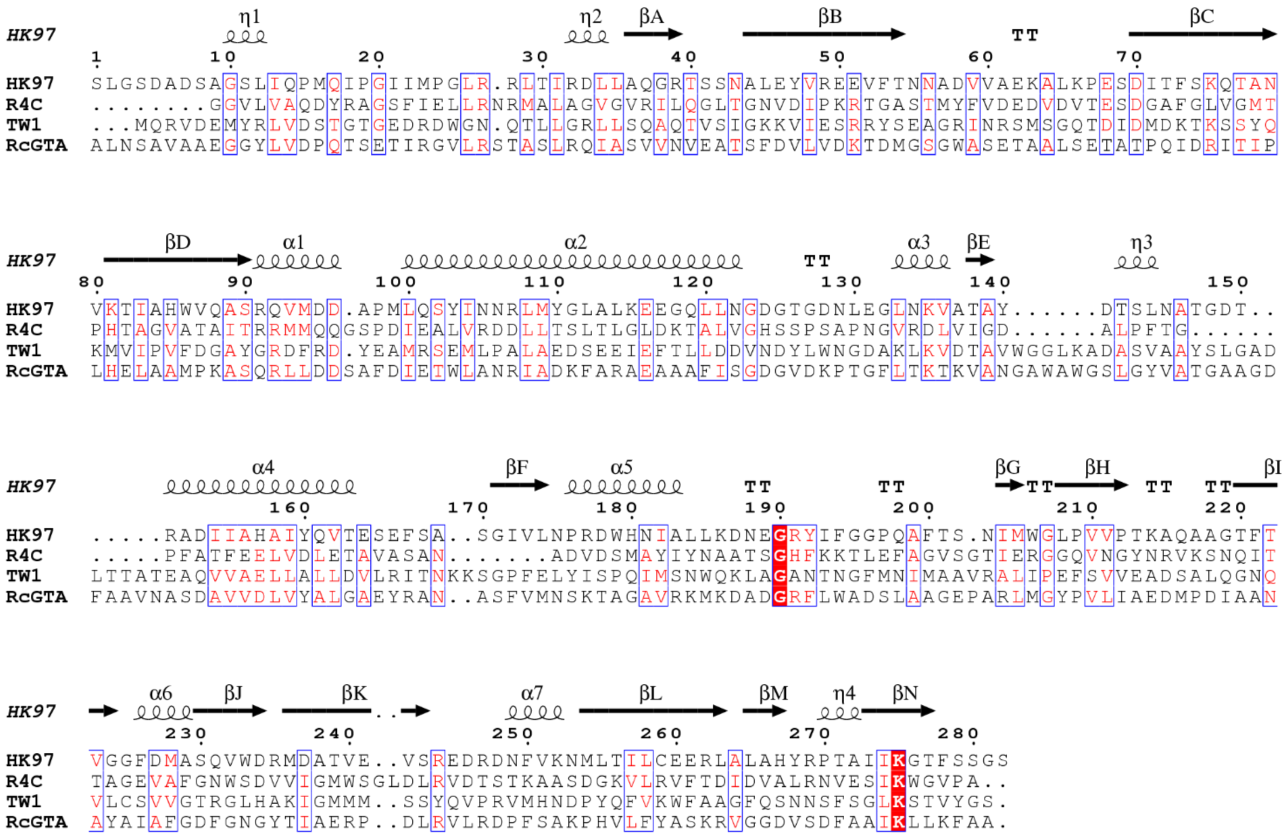

Supplementary Fig. 2. Structure-based multiple sequence alignment of the MCPs of the R4C, HK97, TW1 and RcGTA phages. GenBank accession nos. of MK882925, KC542353, U18319.1, AYPR01000020 for the MCP genes of the R4C, HK97, TW1 phages and RcGTA, respectively, are used for analysis.

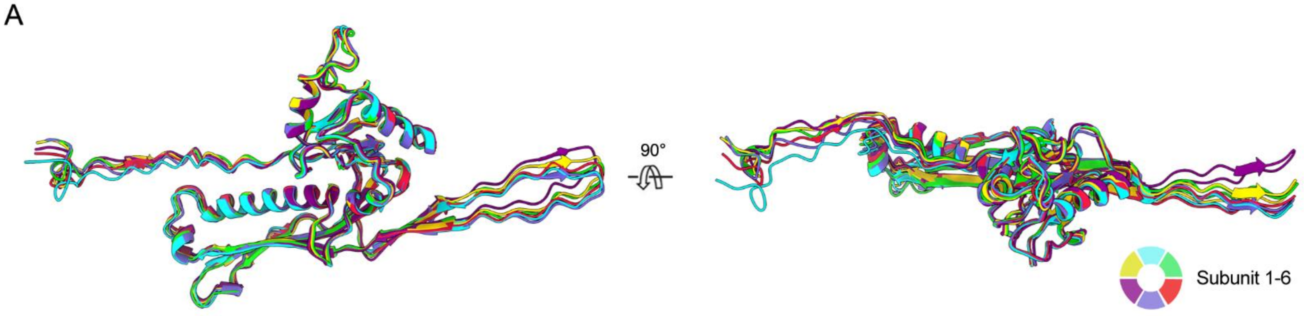

Supplementary Fig. 3. Superimposition of six hexameric MCPs from one hexon. (A) The top (left) and side (right) views are shown and the six subunits are in different color.

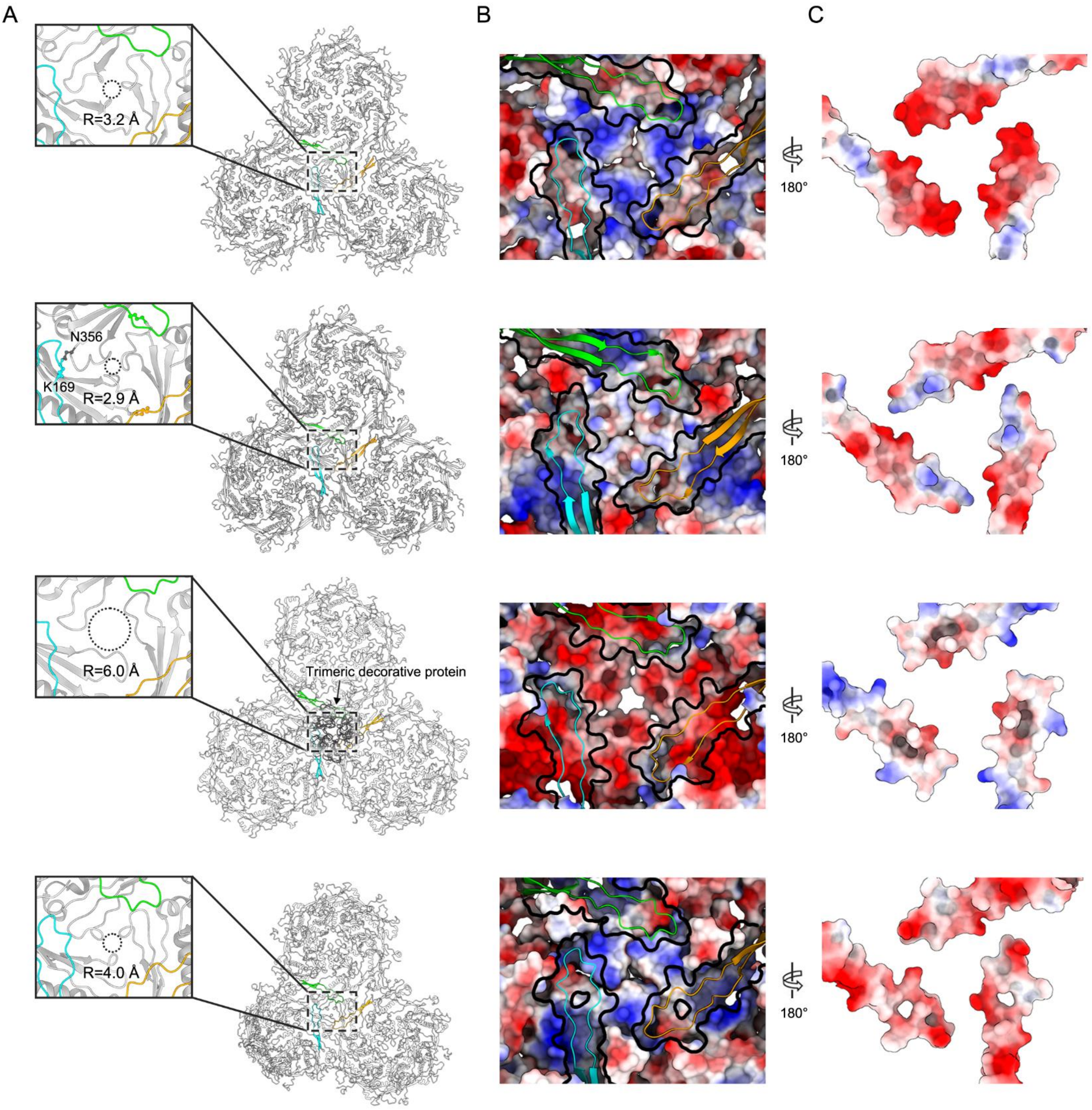

Supplementary Fig. 4. Comparisons of capsomer interactions around the three-fold axis among R4C, HK97 (PDB ID: 1OHG), TW1 (PDB ID: 5WK1) and RcGTA (PDB ID: 6TSU). A. From top to bottom, capsomers are organized around the three-fold axes of R4C, HK97, TW1 and RcGTA. Zoom-in views show the core tunnels formed by the three P-loops. Diameters of the core tunnels are labelled. **B.** Relative electrostatic potential surface representations (except the E-loops, which are shown in cartoon form) around the three-fold axis. E-loops are outlined with black lines. **C.** Electrostatic potential surfaces of representations of E-loops in (**B**) are shown but rotated 180° relative to (**B**).

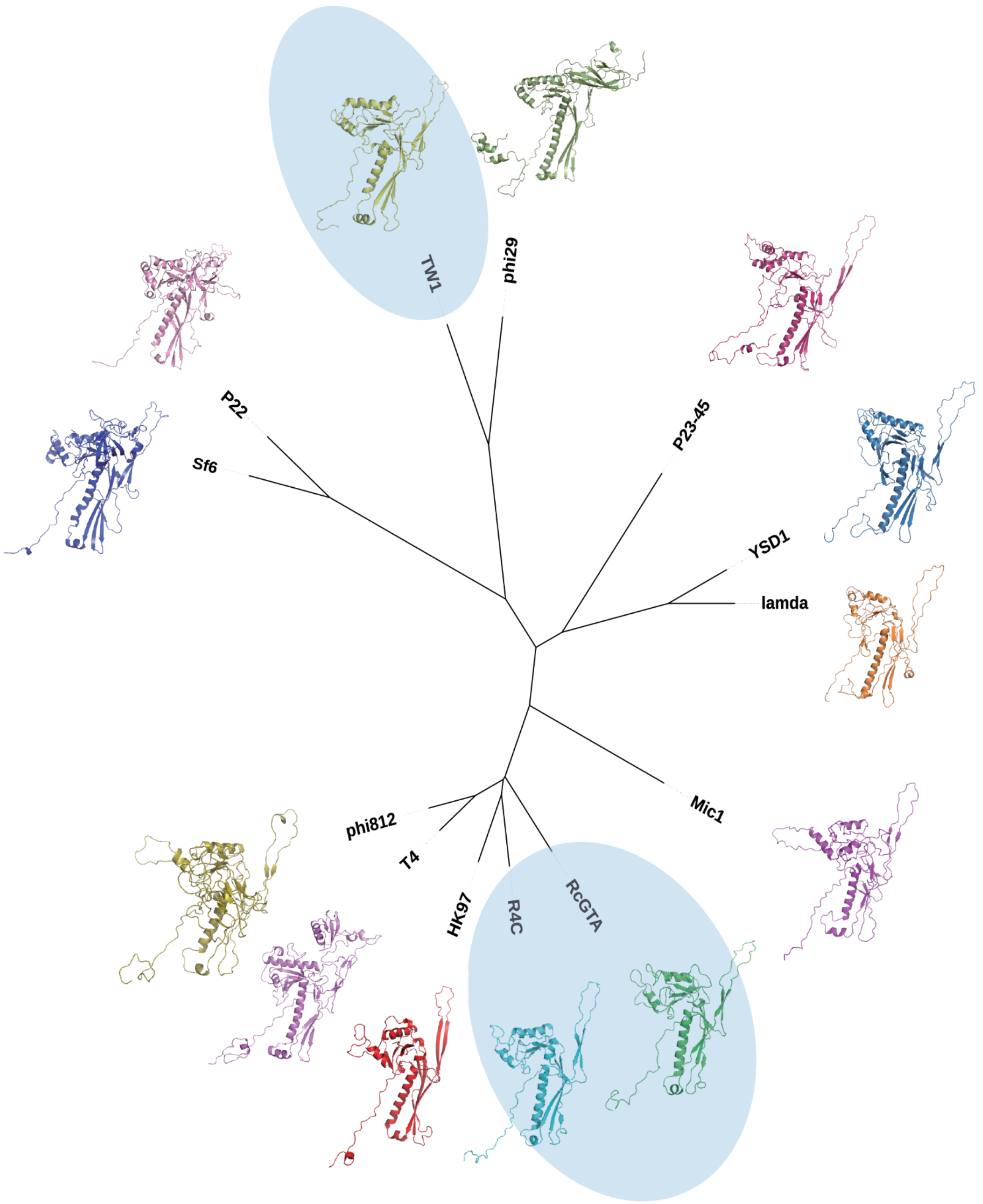

Supplementary Fig. 5. Structure-based phylogenetic tree analysis for the HK97-like fold major capsid proteins of *Caudoviridae* phages. Using PyMol for superposition, the tree shows the major capsid proteins (MCPs) of the following phages (with host and Protein Data Bank codes inside parentheses): phi812 (*Staphylococcus aureus*, 5LII); T4 (*Escherichia coli*, 5VF3); HK97 (*Escherichia coli*, 1OHG); R4C (*D. shibae*); RcGTA (*Rhodobacter capsulatus*, 6TB9); Mic1 (*Microcystis wesenbergii*, 6J3Q); lambda (*Escherichia coli*, 7VIK); YSD1 (*Salmonella enterica*, 6XGQ); P23-45 (*Thermus thermophilus*, 6I9E); phi29 (*Bacillus subtilis*, 6QVK); TW1 (*Pseudoalteromonas phenolica*, 5WK1); P22 (*Salmonella enterica*, 5UU5); Sf6 (*Shigella flexneri*, 5L35). Marine phages are represented by blue shading.

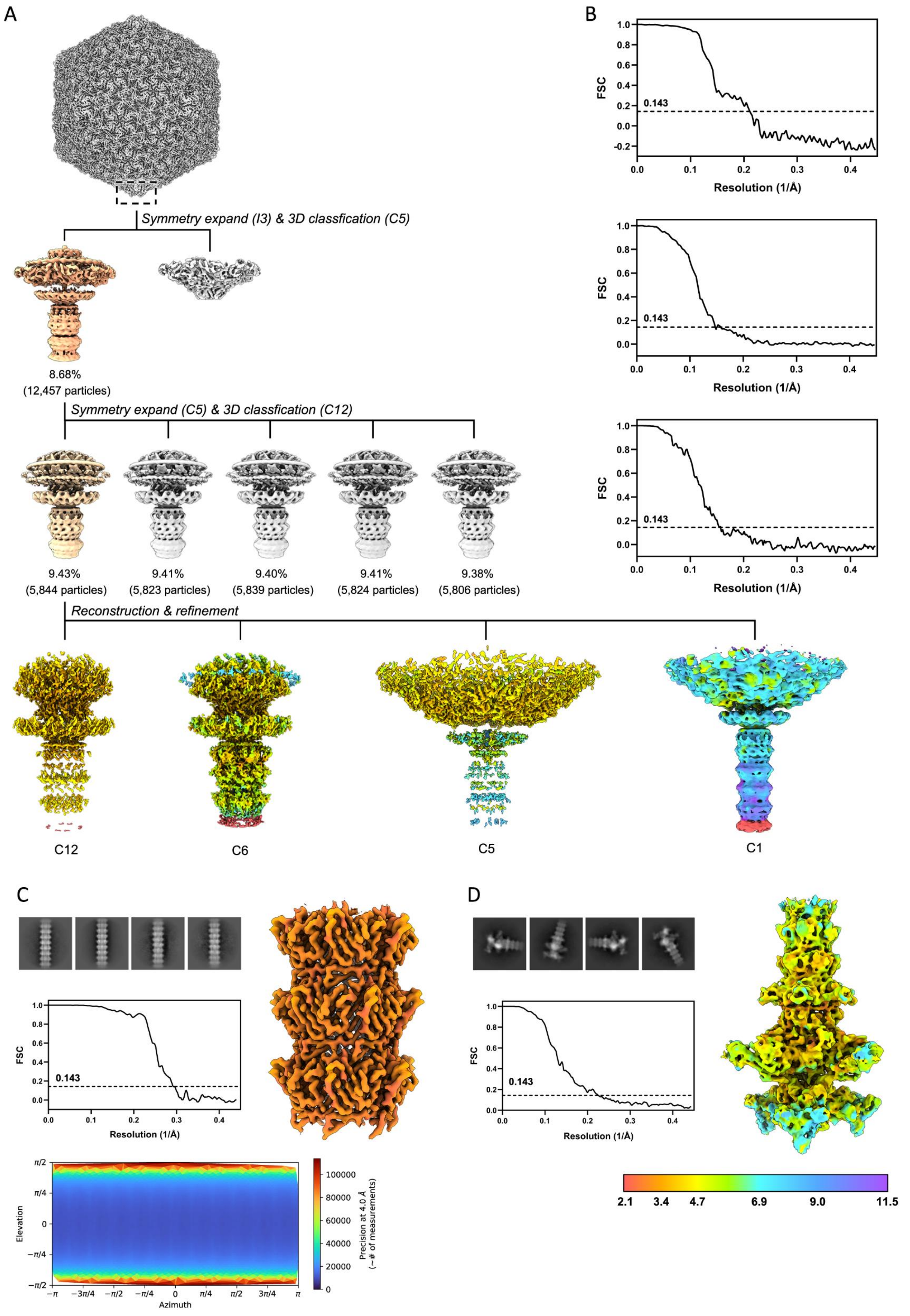

A
Symmetry expand (I3) & 3D classfication (C5)
8.68%
(12,457 particles)
Symmetry expand (C5) & 3D classfication (C12)
9.43%
(5,844 particles)
9.41%
(5,823 particles)
9.40%
(5,839 particles)
9.41%
(5,824 particles)
9.38%
(5,806 particles)
Reconstruction & refinement
C12
C6
C5
C1
B
FSC
0.143
Resolution (1/Å)
C
Elevation
Azimuth
Precision at 4.0 Å
(~# of measurements)
D
2.1
3.4
4.7
6.9
9.0
11.5

Supplementary Fig. 6. Workflow for the reconstruction of the R4C tail. A. Flowchart for 3D classification and refinement of the portal vertex and the head-to-tail connector. The sub-particles of the five-fold vertexes were re-extracted, and the special portal vertexes were subjected to focused classification by imposing five-fold (C5) symmetry. 12,457 particles representing to the portal vertex were then subjected to symmetry expansion (C5) and additional 3D classification by imposing C12 symmetry, thereby obtaining five classes that differed by 72° in relative orientation. Sub-particles from one of the five classes were used to calculate the C1 portal vertex, C5 portal vertex, C6 neck and C12 neck by imposing different symmetries. Final 3D reconstruction maps are shown and are coloured according to local resolution. **B.** Fourier shell correlation (FSC) curves of 3D reconstructions of the C12 (top), C6 (middle) and C5 (bottom) portal vertex. **C, D.** Reconstruction of the tail tube (**C**) and tail end complex (**D**). Representative 2D class averages, FSC curves of 3D reconstructions, and the final 3D density maps (coloured according to local resolution) are shown. The angular distribution of tail tube particles used in the final reconstruction is shown (C).

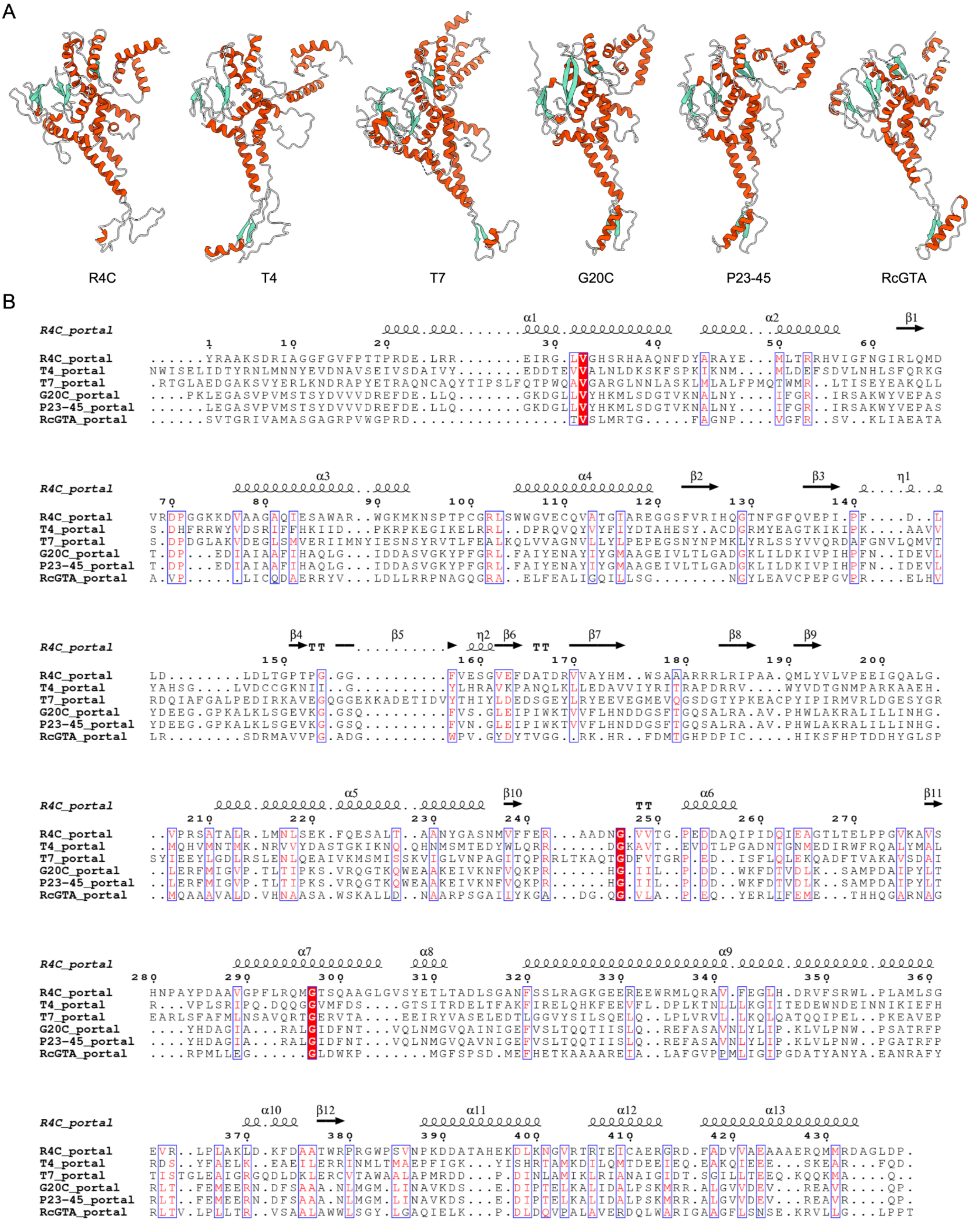

A
R4C
T4
T7
G20C
P23-45
RcGTA
B
R4C_portal α1 α2 β1
1 10 20 30 40 50 60
R4C_portal ......YRAAKSDRIAGGFGVFPTTPRDE.LRR.......EIRG.LVGHSRHAAQNFDYARAYE...MLTRRHVIGFNGIRLQMD
T4_portal NWISELIDTYRNLMNNYEVDNAVSEIVSDAIVY.......EDDTEVVALNLDKSKFSPKIKNM....MLDEFSDVLNHLSFQRKG
T7_portal .RTGLAEDGAKSVYERLKNDRAPYETRAQNCAQYTIPSLFQTPWQAVGARGLNNLASKLMLALFPMQTWMR..LTISEYEAKQLL
G20C_portal ....PKLEGASVPVMSTSYDVVVDREFDE.LLQ.......GKDGLLVYHKMLSDGTVKNALNY....IFGR..IRSAKWYVEPAS
P23-45_portal ......LEGASVPVMSTSYDVVVDREFDE.LLQ.......GKDGLLVYHKMLSDGTVKNALNY....IFGR..IRSAKWYVEPAS
RcGTA_portal ......SVTGRIVAMASGAGRPVWGPRD................TVSLMRTG.....FAGNP....VGFR..SV..KLIAEATA
R4C_portal α3 α4 β2 β3 η1
70 80 90 100 110 120 130 140
R4C_portal VRDPGGKKDVAAGAQIESAWAR..WGKMKNSPTPCGRLSWWGVECQVATGIAREGGSFVRIHQGTNFGFQVEPI.PF....D..L
T4_portal S.DHFRRWYVDSRIFFHKIID...PKRPKEGIKELRRL..DPRQVQYVYFIYDTAHESY.ACDGRMYEAGTKIKIPK....AAVV
T7_portal S.DPDGLAKVDEGLSMVERIIMNYIESNSYRVTLFEALKQLVVAGNVLLYLPEPEGSNYNPMKLYRLSSYVVQRDAFGNVLQMVT
G20C_portal T.DP...EDIAIAAFIHAQLG...IDDASVGKYPFGRL.FAIYENAYIYGMAAGEIVLTLGADGKLILDKIVPIHPFN..IDEVL
P23-45_portal T.DP...EDIAIAAFIHAQLG...IDDASVGKYPFGRL.FAIYENAYIYGMAAGEIVLTLGADGKLILDKIVPIHPFN..IDEVL
RcGTA_portal A.VP.....LICQDAERRYV...LDLLRRPNAGQGRA..ELFEALIGQILLSG........NGYLEAVCPEPGVPR....ELHV
R4C_portal β4 TT β5 η2 β6 TT β7 β8 β9
150 160 170 180 190 200
R4C_portal LD........LDLTGPTPG.GG..........FVESGVEFDATDRVVAYHM..WSAAARRRLRIPAA.QMLYVLVPEEIGQALG.
T4_portal YAHSG....LVDCCGKNII..G..........YLHRAVKPANQLKLLEDAVVIYRITRAPDRRV....WYVDTGNMPARKAAEH.
T7_portal RDQIAFGALPEDIRKAVEGQGGEKKADETIDVYTHIYLDEDSGEYLRYEEVEGMEVQGSDGTYPKEACPYIPIRMVRLDGESYGR
G20C_portal YDEEG.GPKALKLSGEVKG.GSQ.........FVS.GLEIPIWKTVVFLHNDDGSFTGQSALRA.AV.PHWLAKRALILLINHG.
P23-45_portal YDEEG.GPKALKLSGEVKG.GSQ.........FVN.GLEIPIWKTVVFLHNDDGSFTGQSALRA.AV.PHWLAKRALILLINHG.
RcGTA_portal LR........SDRMAVVPG.ADG.........WPV.GYDYTVGG..RK.HR..FDMTGHPDPIC.....HIKSFHPTDDHYGLSP
R4C_portal α5 β10 TT α6 β11
210 220 230 240 250 260 270
R4C_portal ..VPRSATALR.LMNLSEK.FQESALT..AANYGASNMVFFER...AADNG.VVTG.PEDDAQIPIDQIEAGTLTELPPGVKAVS
T4_portal ..MQHVMNTMK.NRVVYDASTGKIKNQ.QHNMSMTEDYWLQRR......DGKAVT..EVDTLPGADNTGNMEDIRWFRQALYMAL
T7_portal SYIEEYLGDLRSLENLQEAIVKMSMISSKVIGLVNPAGITQPRRLTKAQTGDFVTGRP.ED..ISFLQLEKQADFTVAKAVSDAI
G20C_portal ..LERFMIGVP.TLTIPKS.VRQGTKQWEAAKEIVKNFVQKPR......HGIIL..P.DD..WKFDTVDLK..SAMPDAIPYLT
P23-45_portal ..LERFMIGVP.TLTIPKS.VRQGTKQWEAAKEIVKNFVQKPR......HGIIL..P.DD..WKFDTVDLK..SAMPDAIPYLT
RcGTA_portal ..MQAAAVALD.VHNAASA.WSKALLD..NAARPSGAIIYKGA...DG.QG.VLA..P.EQ..YERLIFEME...THHQGARNAG
R4C_portal α7 α8 α9
280 290 300 310 320 330 340 350 360
R4C_portal HNPAYPDAAVGPFLRQMGTSQAAGLGVSYETLTADLSGANFSSLRAGKGEEREEWRMLQRAV.FEGLH.DRVFSRWL.PLAMLSG
T4_portal R...VPLSRIPQ.DQQGGVMFDS....GTSITRDELTFAKFIRELQHKFEEVFL.DPLKTNLLLKGIITEDEWNDEINNIKIEFH
T7_portal EARLSFAFMLNSAVQRTGERVTA....EEIRYVASELEDTLGGVYSILSQELQ..LPLVRVL.LKQLQATQQIPEL..PKEAVEP
G20C_portal ....YHDAGIA...RALGIDFNT...VQLNMGVQAINIGEFVSLTQQTIISLQ..REFASAVNLYLIP.KLVLPNW..PSATRFP
P23-45_portal ....YHDAGIA...RALGIDFNT...VQLNMGVQAVNIGEFVSLTQQTIISLQ..REFASAVNLYLIP.KLVLPNW..PGATRFP
RcGTA_portal ....RPMLLEG......GLDWKP.......MGFSPSD.MEFHETKAAAAREIA..LAFGVPPMLIGIPGDATYANYA.EANRAFY
R4C_portal α10 β12 α11 α12 α13
370 380 390 400 410 420 430
R4C_portal EVR..LPLAKLD.KFDAATWRPRGWPSVNPKDDATAHEKDLKNGVRTRTEICAERGRD.FADVVAEAAAERQMMRDAGLDP.
T4_portal RDS..YFAELK..EAEILERRINMLTMAEPFIGK...YISHRTAMKDILQMTDEEIEQ.EAKQIEEE...SKEAR..FQD.
T7_portal TISTGLEAIGRGQDLDKLERCVTAWAALAPMRDD...P.DINLAMIKLRIANAIGIDT.SGILLTEEQ.KQQKMA....Q..
G20C_portal RLT..FEMEERN.DFSAAA.NLMGM.LINAVKDS...E.DIPTELKALIDALPSKMRR.ALGVVDEV...REAVR....QP.
P23-45_portal RLT..FEMEERN.DFSAAA.NLMGM.LINAVKDS...E.DIPTELKALIDALPSKMRR.ALGVVDEV...REAVR....QP.
RcGTA_portal RLTV.LPLLTR..VSAALAWWLSGY.LGAQIELK...P.DLDQVPALAVERDQLWARIGAAGFLSNSE.KRVLLG...LPPT

Supplementary Fig. 7. The portal proteins among different species share a general topology but low sequence identity. A. Structure comparison of the R4C portal protein and those from other organisms including T4 (PDB ID: 3JA7), T7 (PDB ID: 6QX5), G20C (PDB ID: 6IBG), P23-45 (PDB ID: 6QJT) and RcGTA (PDB ID: 6TE8). Models are shown in ribbon representation with the α-helix and β-sheet coloured in orange-red and turquoise, respectively. **B.** Structure-based sequence alignment of the portal proteins of the R4C, T4, T7, G20C, P23-45 and RcGTA (GenBank accession nos. of MK882925, QZB89778, QZB84878, API81889, YP_001467939.1, AYPR01000020, respectively).

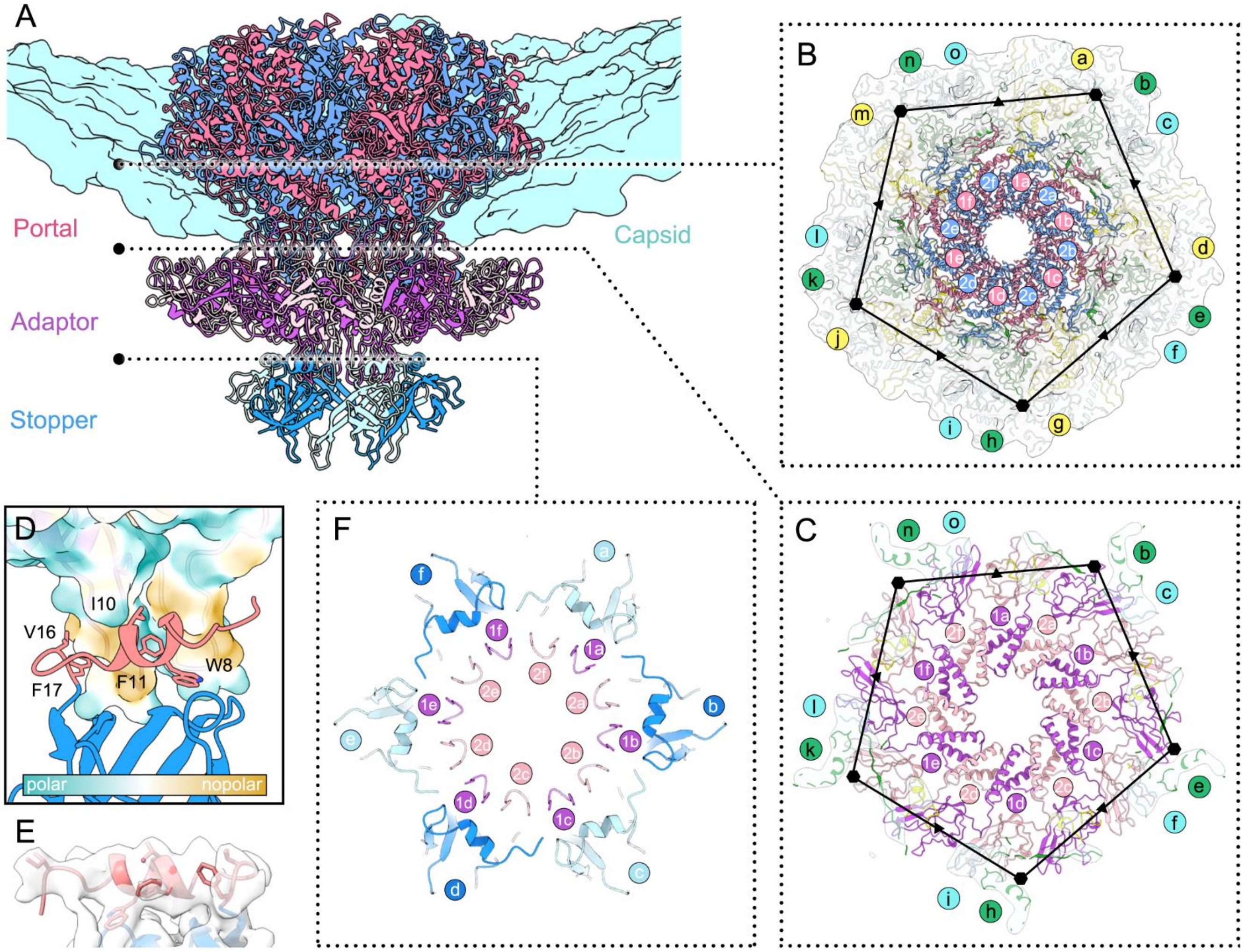

Supplementary Fig. 8. Symmetry mismatch in the phage neck. A. Five-fold icosahedral axis occupied by the head-to-tail connector comprising the C12 portal (blue and deep pink), C12 adaptor (purple and pink) and C6 stopper (blue and light blue). Cartoon models of the head-to-tail connector and the peripheral density map of the capsid (cyan) are shown. **B, C.** Symmetry mismatch between the C5 portal vertex and C12 portal proteins (**B**), and between the C5 portal vertex and C12 adaptor (**C**). Odd and even subunits are labelled clockwise. The major capsid proteins (MCPs, ribbon representation) are fitted into the C5 map (semitransparent surface representation), with the subunits involved in tail binding numbered clockwise and coloured according to three different colours (yellow, green, cyan). Pentagons and triangles show the positions of the 5-fold and 3-fold axes, respectively. **D, E.** Interactions between the adaptor and stopper proteins. Extensive hydrophobic interactions between the N-terminal helix of the stopper proteins (ribbon) and the adaptor loops of the adaptor proteins (surface) are shown in (**D**). The N-terminal helix is highlighted in red with the nonpolar residues represented in stick representation. The adaptor proteins are coloured in a yellow-turquoise gradient (hydrophobic, yellow; hydrophilic, turquoise). The hydrophobic side chains of the stopper N-terminal helix shown in (**D**) fitted well into the corresponding density map (**E**). **F.** Symmetry mismatched between the C12 adaptor and the C6 stopper is mediated by the interface between the loops of the adaptor proteins and the N-terminal helices of the stopper proteins. Odd and even adaptor subunits, and stopper are labelled clockwise.

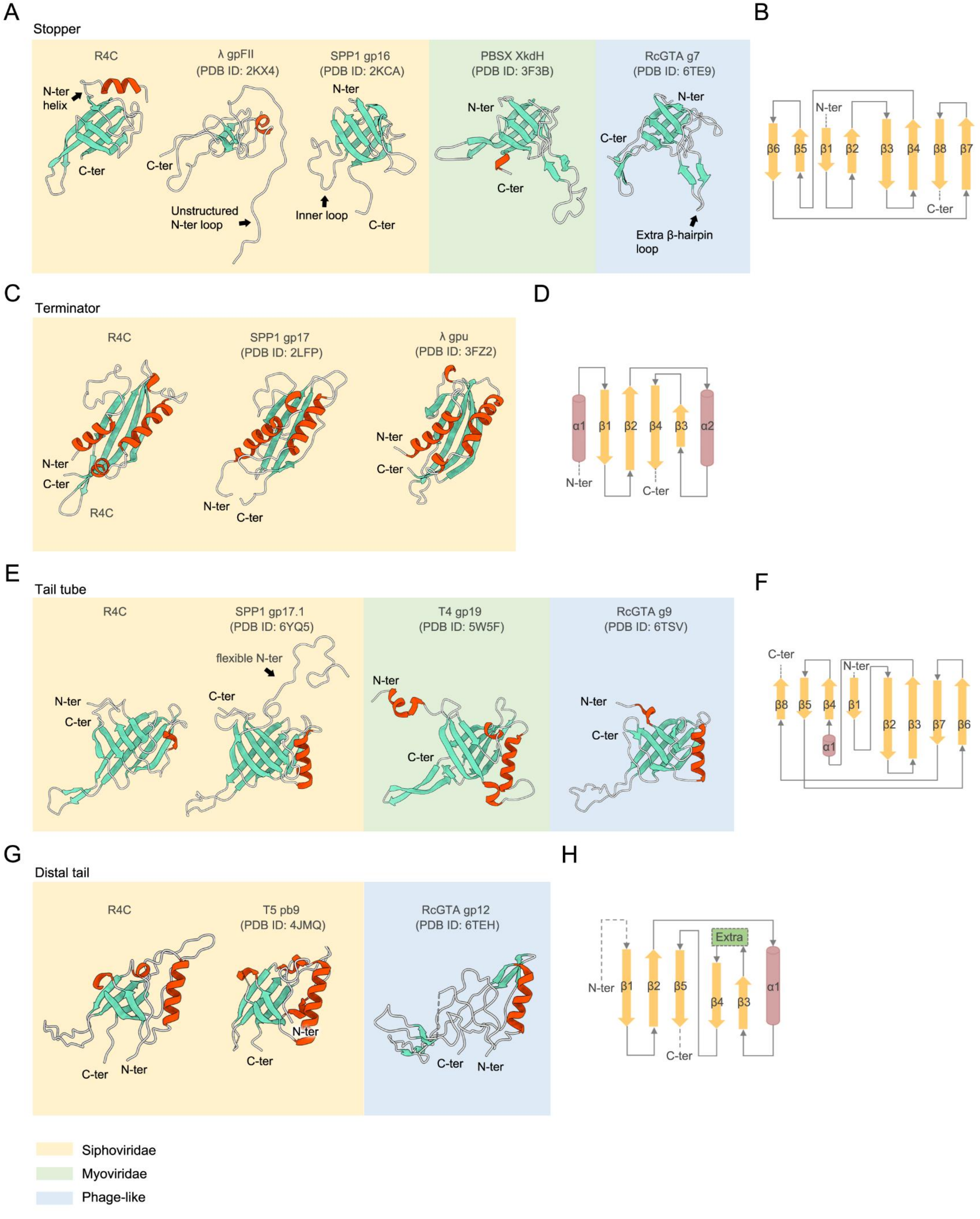
A
Stopper
R4C
N-ter helix
C-ter
λ gpFII
(PDB ID: 2KX4)
C-ter
Unstructured N-ter loop
SPP1 gp16
(PDB ID: 2KCA)
N-ter
Inner loop
C-ter
PBSX XkdH
(PDB ID: 3F3B)
N-ter
C-ter
RcGTA g7
(PDB ID: 6TE9)
N-ter
C-ter
Extra β-hairpin loop
B
β6 β5 β1 β2 β3 β4 β8 β7
N-ter
C-ter
C
Terminator
R4C
N-ter
C-ter
R4C
SPP1 gp17
(PDB ID: 2LFP)
N-ter
C-ter
λ gpu
(PDB ID: 3FZ2)
N-ter
C-ter
D
α1 β1 β2 β4 β3 α2
N-ter
C-ter
E
Tail tube
R4C
N-ter
C-ter
SPP1 gp17.1
(PDB ID: 6YQ5)
flexible N-ter
C-ter
T4 gp19
(PDB ID: 5W5F)
N-ter
C-ter
RcGTA g9
(PDB ID: 6TSV)
N-ter
C-ter
F
C-ter
N-ter
β8 β5 β4 β1 β2 β3 β7 β6
α1
G
Distal tail
R4C
C-ter
N-ter
T5 pb9
(PDB ID: 4JMQ)
N-ter
C-ter
RcGTA gp12
(PDB ID: 6TEH)
C-ter
N-ter
H
N-ter
β1 β2 β5 β4 β3 α1
Extra
C-ter
Siphoviridae
Myoviridae
Phage-like

Supplementary Fig. 9. Morphology and topology of the tail-related proteins show structural diversity and similarity among different species. A, C, E, G. Structural comparison of the R4C tail relative proteins—stopper (**A**), terminator (**C**), tail tube (**E**) and distal tail (**G**)—with corresponding structural homologous proteins from other species. Models are represented as coloured ribbons (α-helix, orange red; β-sheet, turquoise) and are indicated by background colouring according to the family to which they belong: siphophage, yellow; myophage, green; *Phage-like*, blue. **B, D, F, H.** Shared topology diagrams of secondary structure elements for the four types of proteins. Helices and strands are represented by cylinders and arrows, respectively.

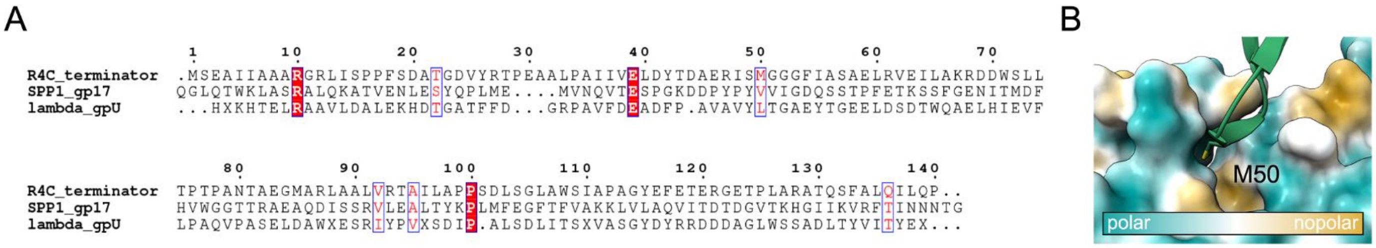

Supplementary Fig. 10. Conserved hydrophobic motifs involved in inter-layer interactions among the R4C, SPP1 and lambda terminator proteins. A. Sequence alignment of the terminator proteins of R4C, SPP1 and λ, suggesting conservation of the hydrophobic motifs near the M50 site. GenBank accession nos. of QDF14266.1, O48448.1 and OYC12327.1 for the genes of the terminator proteins of R4C, SPP1 and λ, respectively, were used in the analysis. **B.** M50 of the R4C terminator protein (green stick) inserts its hydrophobic side chain into the pocket formed by the tail tube proteins. The tail tube proteins are shown in surface representation and are coloured with a yellow-turquoise gradient, where yellow indicates a hydrophobic surface and turquoise, a hydrophilic surface.

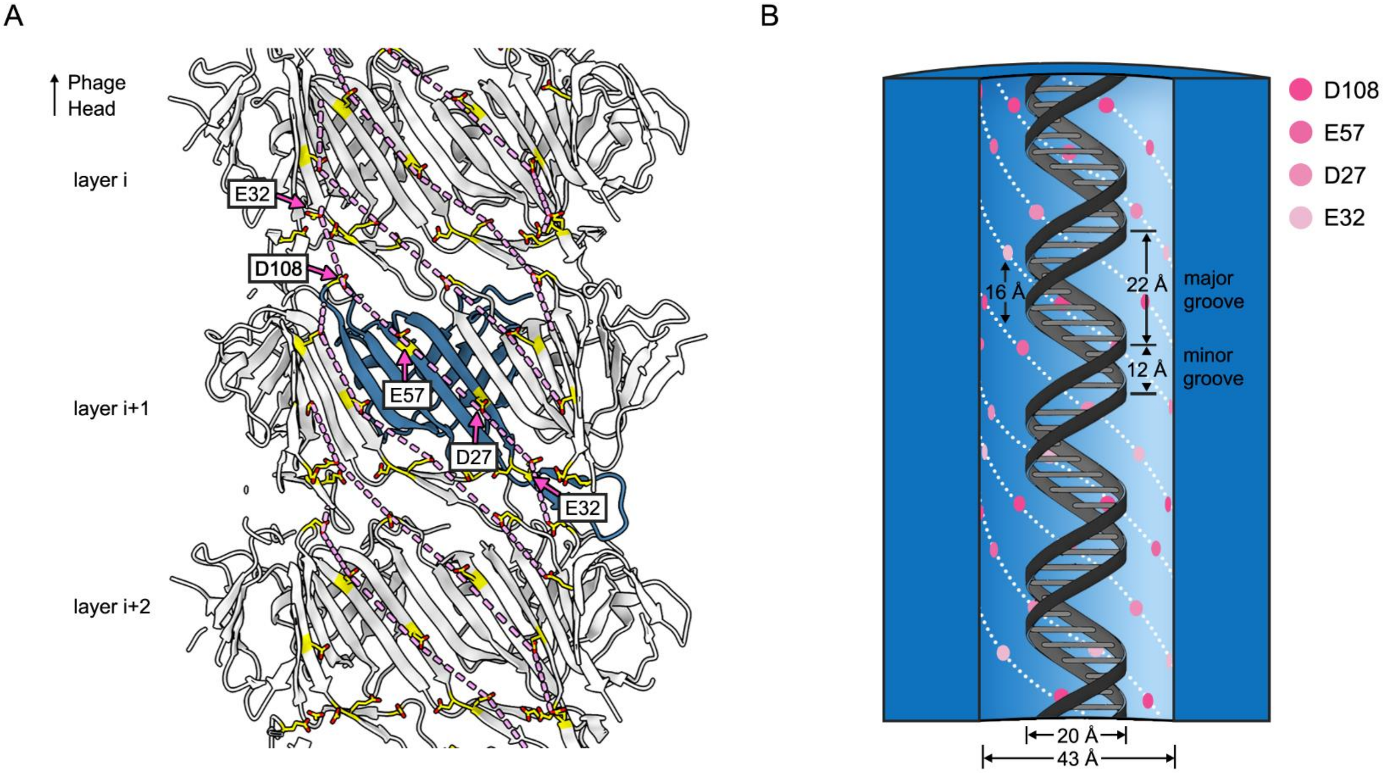

Supplementary Fig. 11. Negative-charged track in the inner surface of the tail tube for DNA translocation. A. Cut-away view of the tail tube. Acidic residues lining the inner surface are represented as yellow sticks, with the imaginary helical tracks demarcated by a dashed pink line. **B.** Schematic diagram of the negatively charged track presenting the theoretical requirement of DNA to ratchet from the tail tube. The tubular tail is shown in blue and DNA in grey. Four key acidic residues are depicted as circles in different shades of pink, presenting the imaginary helical tracks that are shown as dashed white line.

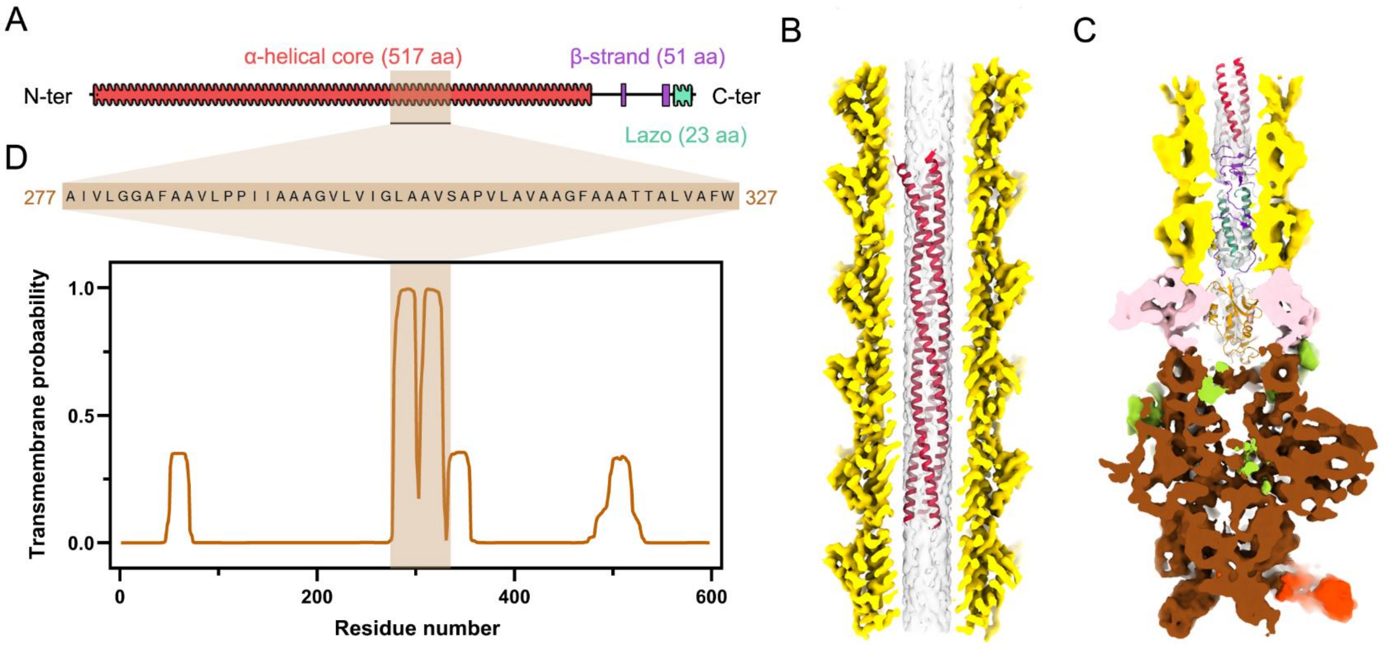

Supplementary Fig. 12. Structural features of the R4C tape measure protein. A. The secondary structure elements of the R4C tape measure protein, shown in jagged lines (α-helices) and broad lines (β-strands) and coloured according to the domains (α-helical core in red; β-strand in purple; Lazo in turquoise). **B, C.** Fitting of the P22 tail-needle protein (PDB ID: 2POH) and R4C peptidase (predicted by trRosetta) into the cryo-EM map of R4C tail. The density for the tape measure protein and peptidase is shown in semi-transparent grey, the tail tube protein in yellow, the distal tail protein in pink, the hub protein in green, the megatron protein in brown. The P22 tail-needle model is colour-coded according to the convention established for R4C in panel (**A**). **D.** The predicted transmembrane region of the R4C tape measure protein highlighted in brown and its corresponding sequence is shown.

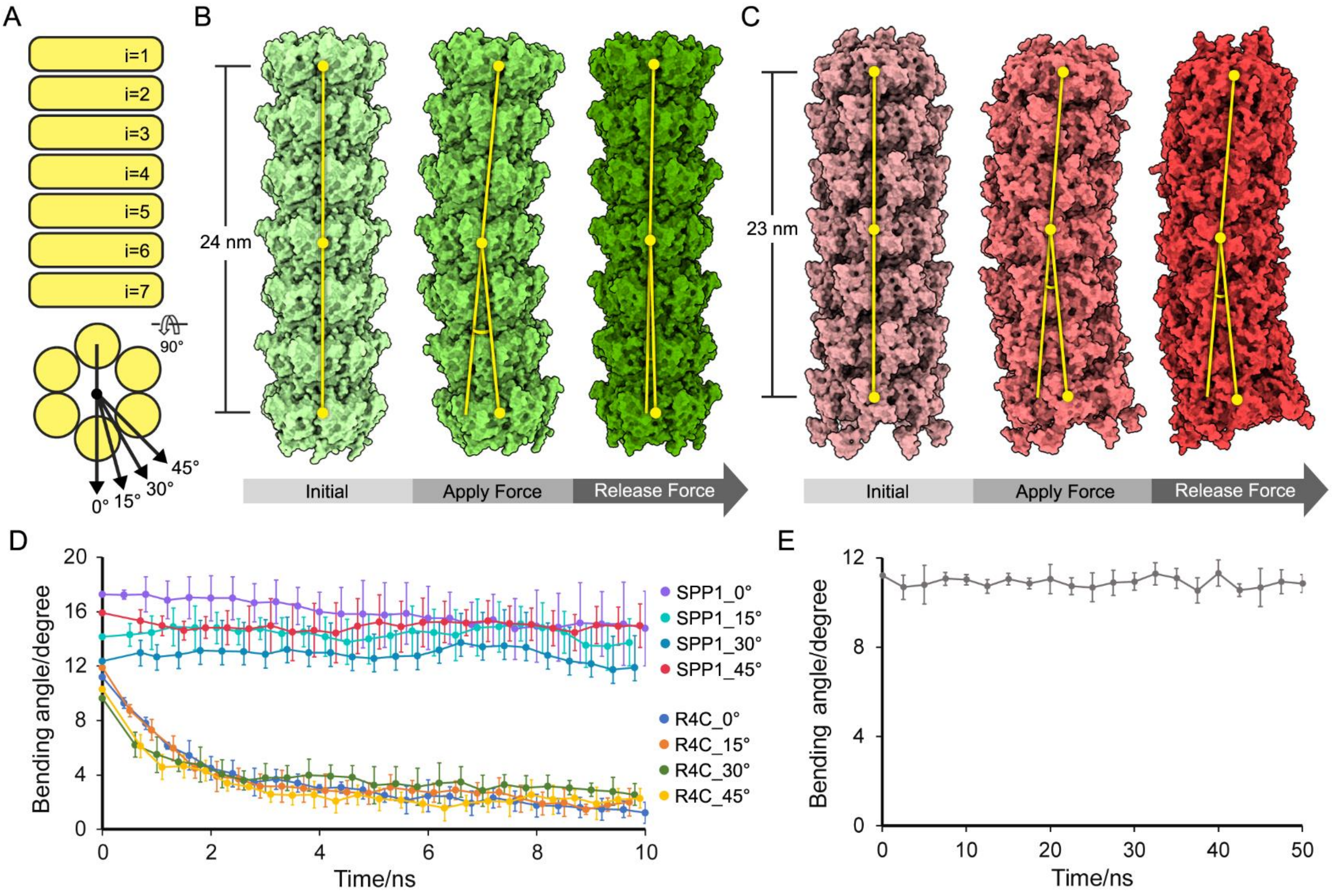

Supplementary Fig. 13. Analysis of tail flexibility of the phage R4C using molecular dynamics (MD) simulation. A. Schematic figure illustrating the steered MD approach, each disc of tube and each monomer in one disc are depicted as yellow rectangle in side-view and as yellow circle in top-view of the tail. White arrows indicate the gradient forces against the tail axis, black narrows indicate the four directions of forces. **B-C.** MD simulation analysis of phage R4C (**B**) and SPP1 (**C**). The tail tube models of the phage R4C and SPP1 were generated during different stages of MD simulations revealed their degree of deformation embodied in the bending angle, different shades of colour correspond to different MD stages. **D**. The bending angle of tail tube from two phages variated over time of MD simulation of unbending process. For clarity, angle data are depicted at intervals of 0.4 ns, and data from four simulations of each phage are spaced by 0.1 ns in trajectory extraction. **E.** The bending angle for R4C tail variated over simulation time by MD based on all-atom structure-based model. The data are depicted at intervals of 2.5 ns. For each bending angle plots (D, E), points represent the mean of five repeated simulations, and error bar indicates the standard deviation.

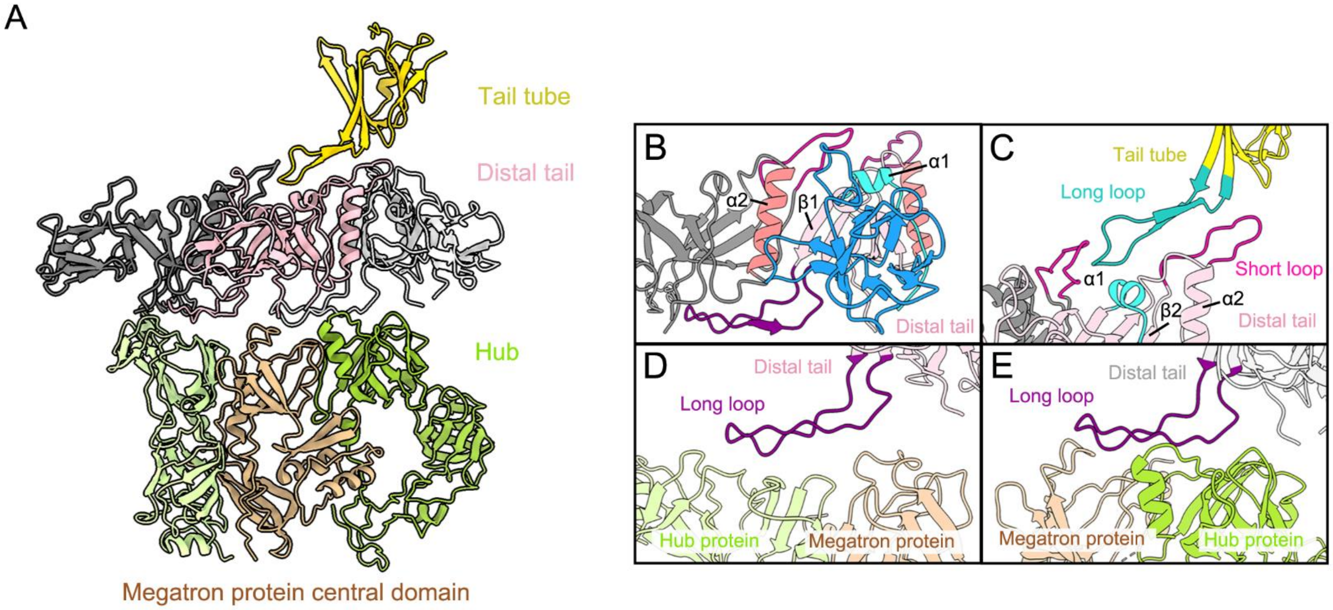

Supplementary Fig. 14. The inter-subunit interface of the distal tail proteins, and the interfaces between distal tail and other tail components. A. One tail tube protein (yellow) interacts with two distal tail proteins (distinguished by deep grey and pink) (see panel [**C**]). Two adjacent distal tail subunits (pink and light grey) interact with two hub proteins (light and dark green) and one central domain of the megatron protein (brown) (see panel [**D, E**]). **B.** The central helix (red) and short loop (deep pink) from one distal tail protein are involved in inter-subunit contacts, and another subunit is coloured by domains: N-terminus (cyan), long loop (purple), central helix (red), insertion domain (blue) and short loop (deep pink). **C.** The contacts between the tail tube and the distal tail are mediated by the long loop (turquoise) of the tail tube protein, short loops (deep pink) from two adjacent distal tail subunits, and the N-terminal helix (cyan) from one distal tail subunit. **D, E.** The long loop of distal tail proteins dominates the asymmetric interactions with the baseplate. The long loop from one distal tail protein makes contact predominantly with the hub protein (**D**). The long loop from another one makes contact with both the megatron protein central domain and the hub protein (**E**).

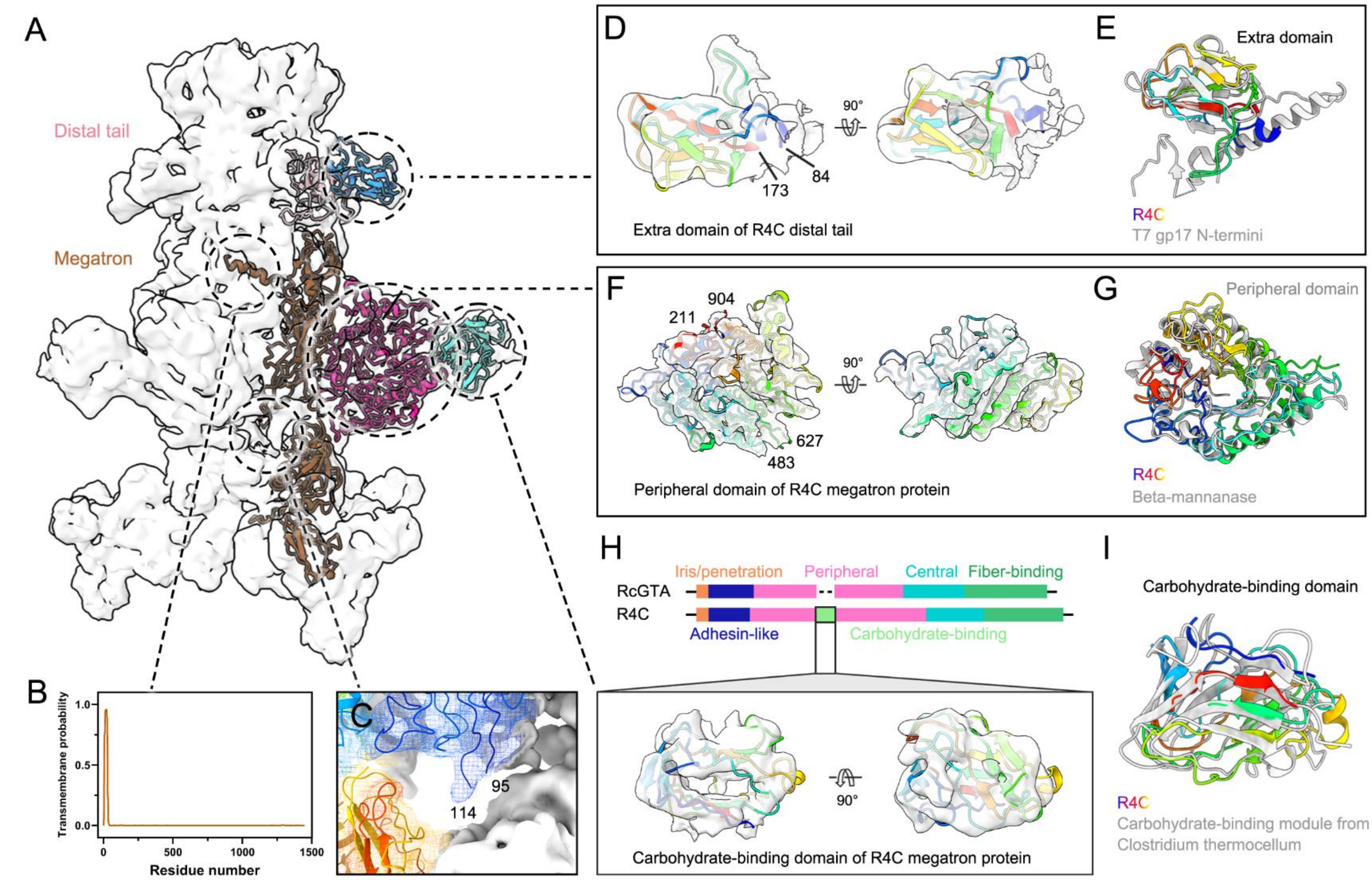

Supplementary Fig. 15. Structural features in the tail end that relate to phage infection. A. Density map of the tail end fitted with a distal tail protein (pink ribbon) and a megatron protein (brown ribbon). The proposed adsorption devices involved in host recognition are highlighted and described in (**D-H**), including the distal tail protein extra domain (blue), the megatron protein peripheral domain (magenta) and the extra domain (turquoise). **B.** Diagram of the probability of forming a transmembrane helix for the N-terminal sequence of the megatron protein. The N-terminal helix may serve as a membrane penetration helix. **C.** Megatron protein model (rainbow ribbon) fitted into the corresponding density map (mesh) to show the flexible regions (95-114). **D.** Top (left) and side (right) views of the density map of the R4C fitting with the corresponding model (represented as a rainbow ribbon). **E.** Superimposition of the R4C distal tail extra domain and the reported T7 gp17 N-termini (PDB ID: 7BOZ). **F.** Top (left) and side (right) views of the density map of the R4C megatron protein peripheral domain fitting with the corresponding model (represented as a rainbow ribbon). **G.** Superimposition of the R4C megatron protein peripheral domain and the reported beta-mannanase BaMan113A (PDB ID: 7DVJ). **H.** The megatron protein of R4C is structurally similar to that of RcGTA but possesses an extra domain in its peripheral domain. Inset shows the top (left) and side (right) views of the model of the extra domain (represented as rainbow ribbon), which is further identified as a carbohydrate-binding domain and fits well into the corresponding density map. **I.** Superimposition of the R4C carbohydrate-binding domain and a reported family 11 carbohydrate-binding module (PDB ID: 2LRP).

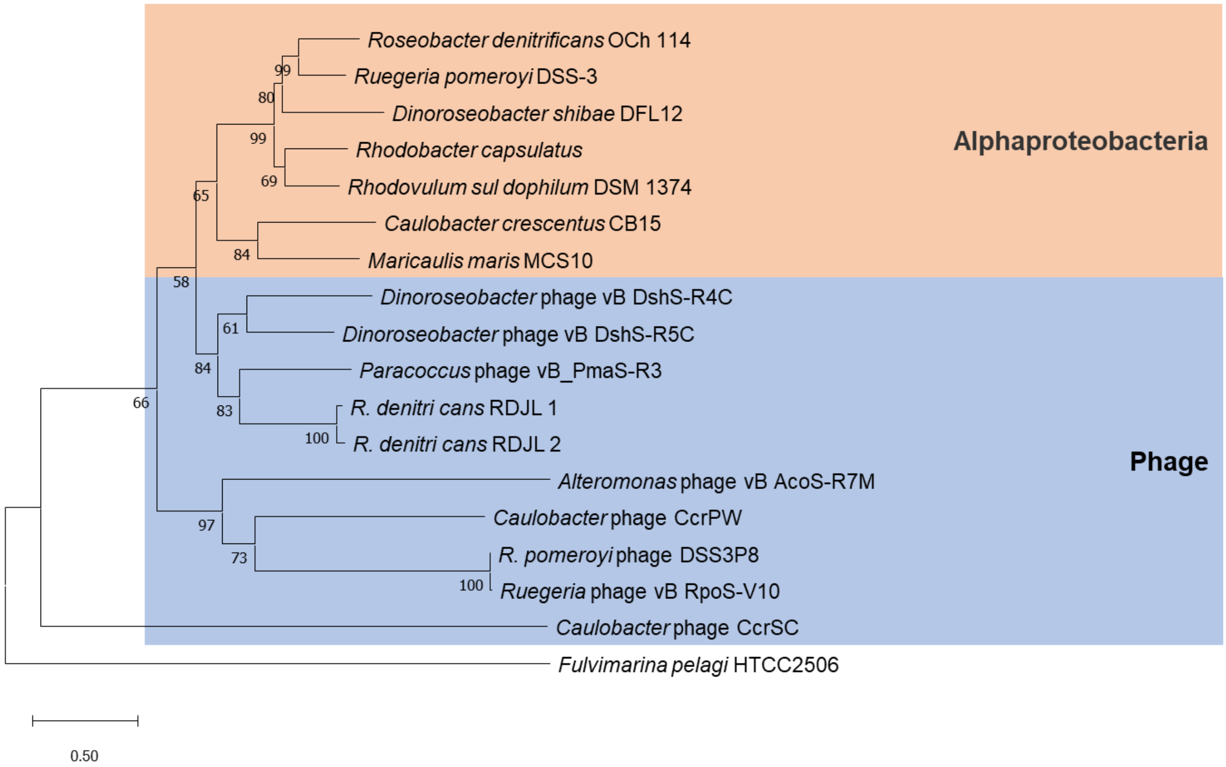

Supplementary Fig. 16. GTA-like baseplate gene cluster (g12/g13/g14/g15) amino acid sequence phylogenetic tree. The GTA-like baseplate gene cluster encodes proteins including the distal tail protein, hub protein, peptidase, and megatron protein. The method of building phylogenetic trees is the maximum adjacency method and bootstrap is 1000.

Supplementary Table 1. Statistics of Cryo-EM data collection, 3D reconstruction, model refinement and model validation.

	Capsid	Portal vertex (C1)	Portal vertex (C5)	Neck (C12)	Neck (C6)	Tail tube	Distal tail and baseplate
**Data Collection and processing**							
Magnification				×93,000			
Microscope				Tecnai F30			
Camera				Falcon3			
Voltage (kV)				300			
Electron exposure dose (e^-/Å^2)				30			
Defocus range (μm)				1.0-2.2			
Pixel size (Å)				1.12			
Micrographs (total)				2,979			
Micrographs (used)	2,979	2,979	2,979	2,979	2,979	2,847	2,912
Final particle images (nos.)	14,272	5,844	5,844	5,844	5,844	103,151	11,598
Symmetry imposed	I2	C1	C5	C12	C6	C6	C3
Map resolution (Å)	3.63	11.50	6.60	4.70	6.60	3.43	4.50
FSC threshold	0.143	0.143	0.143	0.143	0.143	0.143	0.143
Map sharpening B factor (Å^2)	166.7	ND	238.8	250.0	246.5	160.8	124.0
**Database entry**							
EMDB	EMD-34247	EMD-34253	EMD-34254	EMD-34250	EMD-34252	EMD-34248	EMD-34249
PDB	8GTA	\	\	8GTD	8GTF	8GTB	8GTC
**Refinement**							
Model composition							
Non-hydrogen atoms	13,909	\	\	29,592	8,940	17,136	36,150
Protein residues	1,876	\	\	7,392	2,232	2,340	9,033
**R.m.s. deviations**							
RMS (bonds)	0.01	\	\	NA	NA	0.01	NA
RMS (angles)	1.16	\	\	NA	NA	0.67	NA
**Validation**							
MolProbity score	2.16	\	\	NA	NA	1.36	NA
Clashscore	13.98	\	\	NA	NA	5.37	NA
Poor rotamers (%)	0.48	\	\	NA	NA	0.00	NA
**Ramachandran plot**							
Favored (%)	91.51	\	\	NA	NA	97.66	NA
Allowed (%)	8.43	\	\	NA	NA	2.34	NA
Disallowed (%)	0.05	\	\	NA	NA	0.00	NA

NA - not available due to backbone model traced in lower-resolution map; ND – not done.

Supplementary Table 2. Detailed information of structural proteins of Phage R4C.

Protein	Gene name	Total Sequence length	Domain	Sequence	Number of total copies per virion	Symmetry of map	Map resolution (Å)	Modeling building
Adaptor protein	vBDshSR4C_004	178	Attachment	1-106	12	C12	4.7	Backbone model
			Tube	107-130, 149-166				
			Adaptor loop	131-148				
			C-terminal hook	167-178				
Portal protein	vBDshSR4C_005	551	Wing	1-300, 394-475	12	C12	4.7	Backbone model
			Stem	301-323, 377-393				
			Clip	324-376				
			Crown	476-551				
Major capsid protein	vBDshSR4C_006	629	-	-	415	I2	3.63	All-atom model
Stopper protein	vBDshSR4C_009	102	-	-	6	C6	6.6	Backbone model
Tail tube protein	vBDshSR4C_010	130	-	-	120	C6	3.43	All-atom model
Tape measure protein	vBDshSR4C_012	598	-	-	3	-	-	-
Distal tail protein	vBDshSR4C_013	214	-	-	6	C3	4.5	Backbone model
Hub protein	vBDshSR4C_014	291	Attachment	1-141	3	C3	4.5	Backbone model
			Iron-Sulphur cluster	142-161, 244-266				
			Oligosaccharide-binding	162-243				
			Clip	267-291				
Peptidase	vBDshSR4C_015	203	-	-	1	-	-	-
Megatron protein	vBDshSR4C_016	1447	Iris/penetration	1-45	3	C3	4.5	Backbone model
			Adhesion-like	46-211				
			Peripheral	212-481, 630-903				
			Carbohydrate-binding	482-629				
			Central	904-1130				
			Fiber-binding	1131-1447				
Fiber protein	vBDshSR4C_017	230	Rod	1-35	9	C3	4.5	Backbone model
			Knob	36-117				
			Foot	118-230				
Terminator protein	vBDshSR4C_018	140	-	-	6	C6	6.6	Backbone model
