# Supplementary material for: Structure and proposed DNA delivery mechanism of a marine roseophage
Source: Nat Commun. 2023 Jun 17;14:3609. doi: 10.1038/s41467-023-39220-y (PMC10276861; doi:10.1038/s41467-023-39220-y)
Supplement: Supplementary file 3 — Reporting Summary [file 41467_2023_39220_MOESM3_ESM.pdf]

nature portfolio

Corresponding author(s): Ningshao Xia

Last updated by author(s): May 19, 2023

Reporting Summary

Nature Portfolio wishes to improve the reproducibility of the work that we publish. This form provides structure for consistency and transparency in reporting. For further information on Nature Portfolio policies, see our Editorial Policies and the Editorial Policy Checklist.

Statistics

For all statistical analyses, confirm that the following items are present in the figure legend, table legend, main text, or Methods section.

n/a	Confirmed	
☐	☒	The exact sample size (*n*) for each experimental group/condition, given as a discrete number and unit of measurement
☐	☒	A statement on whether measurements were taken from distinct samples or whether the same sample was measured repeatedly
☒	☐	The statistical test(s) used AND whether they are one- or two-sided<br>*Only common tests should be described solely by name; describe more complex techniques in the Methods section.*
☒	☐	A description of all covariates tested
☒	☐	A description of any assumptions or corrections, such as tests of normality and adjustment for multiple comparisons
☐	☒	A full description of the statistical parameters including central tendency (e.g. means) or other basic estimates (e.g. regression coefficient) AND variation (e.g. standard deviation) or associated estimates of uncertainty (e.g. confidence intervals)
☒	☐	For null hypothesis testing, the test statistic (e.g. *F*, *t*, *r*) with confidence intervals, effect sizes, degrees of freedom and *P* value noted<br>*Give P values as exact values whenever suitable.*
☒	☐	For Bayesian analysis, information on the choice of priors and Markov chain Monte Carlo settings
☒	☐	For hierarchical and complex designs, identification of the appropriate level for tests and full reporting of outcomes
☒	☐	Estimates of effect sizes (e.g. Cohen's *d*, Pearson's *r*), indicating how they were calculated

Our web collection on statistics for biologists contains articles on many of the points above.

Software and code

Policy information about availability of computer code

Data collection	CryoEM: The commercial software EPU (vl.11).<br>Molecular dynamics simulation: NAMD (v2.13) and Gromacs (v4.6.7)

Data analysis

1. Phenix (v1.20-4459) (https://www.phenix-online.org);
2. COOT (v0.9) (http://www2.mrc-lmb.cam.ac.uk/personal/pemsley/coot);
3. MotionCor2 (http://msg.ucsf.edu/em/software/motioncor2.html);
4. Gctf (https://en.wikibooks.org/w/index.php?title=Software_Tools_For_Molecular_Microscopy&stable=0#Gctf);
5. CryoSPARC (https://cryosparc.com);
6. Relion (v3.1) (https://relion.readthedocs.io/en/release-3.1/index.html);
7. ResMap (https://resmap.sourceforge.net/#);
8. Discovery Studio (v2017R2) (https://www.3dsbiovia.com/);
9. Chimera (v1.16) (https://www.cgl.ucsf.edu/chimera/);
10. ChimeraX (v1.3) (https://www.cgl.ucsf.edu/chimerax/);
11. VMD (v1.9.2) (https://tcbg.illinois.edu/Research/);
12. trRosetta (https://yanglab.nankai.edu.cn/trRosetta/);
13. Clustal Omega (https://www.ebi.ac.uk/Tools/msa/clustalo/);
14. DALI (http://ekhidna2.biocenter.helsinki.fi/dali/);
15. Molprobity (v4.1) (http://molprobity.manchester.ac.uk/);
16. PyMol (v2.3) (https://pymol.org/2/);
17. ESPript (v3.0) (https://espript.ibcp.fr/ESPript/ESPript/).

For manuscripts utilizing custom algorithms or software that are central to the research but not yet described in published literature, software must be made available to editors and reviewers. We strongly encourage code deposition in a community repository (e.g. GitHub). See the Nature Portfolio guidelines for submitting code & software for further information.

Data

Policy information about availability of data

All manuscripts must include a data availability statement. This statement should provide the following information, where applicable:

- Accession codes, unique identifiers, or web links for publicly available datasets
- A description of any restrictions on data availability
- For clinical datasets or third party data, please ensure that the statement adheres to our policy

The cryo-EM density maps have been deposited in the Electron Microscopy Data Bank (EMDB) with the accession codes of EMD-34247 (capsid) [https://www.ebi.ac.uk/pdbe/entry/emdb/EMD-34247], EMD-34253 (Cl portal vertex) [https://www.ebi.ac.uk/pdbe/entry/emdb/EMD-34253], EMD-34254 (CS portal vertex) [https://www.ebi.ac.uk/pdbe/entry/emdb/EMD-34254], EMD-34250 (Cl2 portal vertex) [https://www.ebi.ac.uk/pdbe/entry/emdb/EMD-34250], EMD-34252 (C6 stopper-terminator) [https://www.ebi.ac.uk/pdbe/entry/emdb/EMD-34252], EMD-34248 (tail tube) [https://www.ebi.ac.uk/pdbe/entry/emdb/EMD-34248], EMD-34249 (distal tail and baseplate) [https://www.ebi.ac.uk/pdbe/entry/emdb/EMD-34249], and the corresponding atomic coordinates have been deposited in the Protein Data Bank (PDB) with the accession codes of 8GTA (capsid) [https://doi.org/10.2210/pdb8gta/pdb], 8GTD (C12 portal-adaptor) [https://doi.org/10.2210/pdb8gtd/pdb], 8GTF (C6 stopper-terminator) [https://doi.org/10.2210/pdb8gtf/pdb], 8GTB (tail tube) [https://doi.org/10.2210/pdb8gtb/pdb], 8GTC (distal tail and baseplate) [https://doi.org/10.2210/pdb8gtc/pdb]. The source data for Supplementary Fig. 13D and 13E are provided as a Source Data file. The MD simulation data generated in this study have been deposited in the Zenodo OpenAIRE database under accession code 7947658 [https://zenodo.org/record/7947658#.ZGXoe6VBz9Y].

Atomic coordinates of previously determined structures are available in the PDB under the following accession codes: 1OHG [https://doi.org/10.2210/pdb1ohg/pdb], 5WK1 [https://doi.org/10.2210/pdb5wk1/pdb], 6TSU [https://doi.org/10.2210/pdb6tsu/pdb], 5LII [https://doi.org/10.2210/pdb5lii/pdb], 5VF3 [https://doi.org/10.2210/pdb5vf3/pdb], 6TB9 [https://doi.org/10.2210/pdb6tb9/pdb], 6J3Q [https://doi.org/10.2210/pdb6j3q/pdb], 7VIK [https://doi.org/10.2210/pdb7vik/pdb], 6XGQ [https://doi.org/10.2210/pdb6xgq/pdb], 6I9E [https://doi.org/10.2210/pdb6i9e/pdb], 6QVK [https://doi.org/10.2210/pdb6qvk/pdb], 5UU5 [https://doi.org/10.2210/pdb5uu5/pdb], 5L35 [https://doi.org/10.2210/pdb5l35/pdb], 3JA7 [https://doi.org/10.2210/pdb3ja7/pdb], 6QX5 [https://doi.org/10.2210/pdb6qx5/pdb], 6IBG [https://doi.org/10.2210/pdb6ibg/pdb], 6QJT [https://doi.org/10.2210/pdb6qjt/pdb], 6TE8 [https://doi.org/10.2210/pdb6te8/pdb], 2KX4 [https://doi.org/10.2210/pdb2kx4/pdb], 2KCA [https://doi.org/10.2210/pdb2kca/pdb], 3F3B [https://doi.org/10.2210/pdb3f3b/pdb], 6TE9 [https://doi.org/10.2210/pdb6te9/pdb], 2LFP [https://doi.org/10.2210/pdb2lfp/pdb], 3FZ2 [https://doi.org/10.2210/pdb3fz2/pdb], 6YQ5 [https://doi.org/10.2210/pdb6yq5/pdb], 5W5F [https://doi.org/10.2210/pdb5w5f/pdb], 6TSV [https://doi.org/10.2210/pdb6tsv/pdb], 4JMQ [https://doi.org/10.2210/pdb4jmq/pdb], 6TEH [https://doi.org/10.2210/pdb6teh/pdb], 2POH [https://doi.org/10.2210/pdb2poh/pdb], 7BOZ [https://doi.org/10.2210/pdb7boz/pdb], 7DVJ [https://doi.org/10.2210/pdb7dvj/pdb], 2LRP [https://doi.org/10.2210/pdb2lrp/pdb].

Research involving human participants, their data, or biological material

Policy information about studies with human participants or human data. See also policy information about sex, gender (identity/presentation), and sexual orientation and race, ethnicity and racism.

Reporting on sex and gender	N/A
Reporting on race, ethnicity, or other socially relevant groupings	N/A
Population characteristics	N/A
Recruitment	N/A
Ethics oversight	N/A

Note that full information on the approval of the study protocol must also be provided in the manuscript.

Field-specific reporting

Please select the one below that is the best fit for your research. If you are not sure, read the appropriate sections before making your selection.

- [x] Life sciences
- [ ] Behavioural & social sciences
- [ ] Ecological, evolutionary & environmental sciences

For a reference copy of the document with all sections, see nature.com/documents/nr-reporting-summary-flat.pdf

Life sciences study design

All studies must disclose on these points even when the disclosure is negative.

Sample size	No statistical method was used to determine the sample size. For cryo-EM analysis, sample size was determined by available microscope time and the number of particles on each micrograph obtained during data collection. 2,979 movies in total were collected and number of particles used for reconstruction is listed in Supplementary table 1. For MD simulation, number of systems and simulation sampling is enough for analysis performed in this study.
Data exclusions	Cryo-EM particles which have poor qualities or are not interested targets were excluded during 2D and 3D classification.
Replication	Experiments were repeated independently at least three times. Replicate experiments were successful.<br>For MD simulation (CMD and SMOG-based MD), we performed five replicated runs. Statistics from all simulations were used for analysis.
Randomization	For cryo-EM, randomization is not relevant, since the single-particle cryo-EM analysis is based on randomly distributed particle subsets and particles for 3D reconstruction were randomly assigned to calculate gold-standard FSC.<br>For MD, randomization do not change our results.<br>The biochemical experiments were not randomized as this study did not allocate experimental groups.
Blinding	Blinding is not relevant to this study as no subjective allocation was involved.

Reporting for specific materials, systems and methods

We require information from authors about some types of materials, experimental systems and methods used in many studies. Here, indicate whether each material, system or method listed is relevant to your study. If you are not sure if a list item applies to your research, read the appropriate section before selecting a response.

Materials & experimental systems

n/a	Involved in the study	
☒	☐	Antibodies
☒	☐	Eukaryotic cell lines
☒	☐	Palaeontology and archaeology
☒	☐	Animals and other organisms
☒	☐	Clinical data
☒	☐	Dual use research of concern
☒	☐	Plants

Methods

n/a	Involved in the study	
☒	☐	ChIP-seq
☒	☐	Flow cytometry
☒	☐	MRI-based neuroimaging
